# Flooding regimes increase avian predation on wildlife prey in tidal marsh ecosystems

**DOI:** 10.1002/ece3.4792

**Published:** 2019-01-13

**Authors:** Karen M. Thorne, Kyle A. Spragens, Kevin J. Buffington, Jordan A. Rosencranz, John Takekawa

**Affiliations:** ^1^ Western Ecological Research Center U.S. Geological Survey Davis California; ^2^ Present address: WRA, Inc. San Rafael California; ^3^ Present address: Suisun Resource Conservation District Suisun City California; ^4^ Present address: Washington Department of Fish and Wildlife Olympia Washington

**Keywords:** ardeids, raptors, San Francisco Bay, sea‐level rise, storms, tides

## Abstract

Within isolated and fragmented populations, species interactions such as predation can cause shifts in community structure and demographics in tidal marsh ecosystems. It is critical to incorporate species interactions into our understanding when evaluating the effects of sea‐level rise and storm surges on tidal marshes. In this study, we hypothesize that avian predators will increase their presence and hunting activities during high tides when increased inundation makes their prey more vulnerable. We present evidence that there is a relationship between tidal inundation depth and time of day on the presence, abundance, and behavior of avian predators. We introduce predation pressure as a combined probability of predator presence related to water level. Focal surveys were conducted at four tidal marshes in the San Francisco Bay, California where tidal inundation patterns were monitored across 6 months of the winter. Sixteen avian predator species were observed. During high tide at Tolay Slough marsh, ardeids had a 29‐fold increase in capture attempts and 4 times greater apparent success rate compared with low tide. Significantly fewer raptors and ardeids were found on low tides than on high tides across all sites. There were more raptors in December and January and more ardeids in January than in other months. Ardeids were more prevalent in the morning, while raptors did not exhibit a significant response to time of day. Modeling results showed that raptors had a unimodal response to water level with a peak at 0.5 m over the marsh platform, while ardeids had an increasing response with water level. We found that predation pressure is related to flooding of the marsh surface, and short‐term increases in sea levels from high astronomical tides, sea‐level rise, and storm surges increase vulnerability of tidal marsh wildlife.

## 1 INTRODUCTION

Sea‐level rise and extreme storm events can alter habitat availability in tidal ecosystems leading to alterations in biological interactions across fauna. Flooding can directly affect temperate tidal marsh habitats and the wildlife populations dependent upon them (Takekawa et al., 2006; Thorne, Buffington, Swanson, & Takekawa, 2013), but little is known about secondary community effects such as predator–prey relationships. Recent projections in sea‐level rise are beginning to include low‐probability but high impact flooding events from storms (Wahl et al., 2017) that are important for understanding impacts on wildlife populations in the coastal zone (Dangendorf et al., 2017; Ummenhofer & Meehl, 2017). Many coastal habitats have been eliminated and fragmented due to human land conversion to urban and agricultural landscapes, resulting in many wildlife species of conservation concern (Monroe et al., 1999). Physical wetland processes and impacts from sea‐level rise are often the focus of climate change vulnerability studies (Albano, Dettinger, & Soulard, 2017; Barnard et al., 2017; Kirwan, Temmerman, Skeehan, Guntenspergen, & Fagherazzi, 2016); however, predator–prey interactions may have a disproportionate, although currently poorly understood, effect on sensitive terrestrial and aquatic communities that reside in the tidal zone (Greenberg, Maldonado, Droege, & McDonald, 2006; Thorne, Takekawa, & Elliott‐Fisk, 2012; Zhang & Gorelick, 2014).

Population dynamics depend on the habitats that a species occupies; thus, changes in habitat availability during flood events can alter population dynamics of tidal marsh species. Predation pressure has been suggested to be especially important in tidal marshes where endemic terrestrial tidal marsh species can be “pushed out” of their habitats when they are flooded by astronomical tides or storm surges (Evens & Page, 1986). Tidal marshes provide habitat for a variety of terrestrial species that use the vegetation for foraging, nesting, and cover from predation; top predators are typically avian species that forage on aquatic fish, larvae, crustaceans, small birds, and mammals (Takekawa et al., 2011). Although endemic species are adapted to living in this tidally dynamic habitat, many species can be sensitive to changes in flooding patterns. For example, the seaside sparrow (*Ammodramus maritimus*) experiences greater risks of nest failure due to predation from various avian and mammalian species when forced to nest at greater heights in the salt marsh (Hunter, 2017). Following extreme weather events, shifts in abundance and behavior of species can be rapid and difficult to detect, especially in small populations of species with low dispersal (Takekawa et al., 2015). Additionally, since extreme events are rare, most studies of biological response are anecdotal and provide a weak attribution to high water levels (van de Pol, Jenouvrier, Cornelissen, & Visser, 2017), but as extreme water levels become more common with warming ocean and atmospheric conditions as predicted, changes in behavioral relationships of wildlife may become common as well (Palmer et al., 2017; Wingfield et al., 2017). Therefore, increases in tidal marsh flooding depth and duration due to sea‐level rise and changes in the magnitude and frequency of storms may alter predation pressure, and change predator–prey relationships in both aquatic and terrestrial marsh systems (Traill et al., 2011).

Habitat availability for terrestrial tidal marsh wildlife depends on the spatial and temporal dynamics of tidal inundation, which are controlled by marsh elevation, location within the tidal prism, complexity of internal channel networks, proximity to hard edges at levees, and marsh vegetation (Overton, Casazza, Takekawa, Strong, & Holyoak, 2014). These characteristics influence the plant community and habitat structure (Pennings & Callaway, 1992; Silvestri, Defina, & Marani, 2005), thereby shaping the availability of habitat resources to wildlife and exposure of many to predators. During high tides, terrestrial marsh wildlife may temporarily move to higher ground (e.g., levees or uplands) or take cover in taller vegetation, behaviors that likely increase their exposure to predators (Evens & Page, 1986), although the specifics are not well understood (Bias & Morrison, 1999). Coincidentally, increases in tidal flooding have been shown to facilitate foraging opportunities for snowy egrets (*Egretta thula*) and great egrets (*Casmerodius albus*), which feed mostly on fish and invertebrates in shallow water, often in tidal marshes (Erwin, 1985).

Dense human populations around estuaries have caused drastic changes to ecosystem functions and have fragmented or altered wildlife habitats, often resulting in small habitat patches (Barbier et al., 2011; Cardinale et al., 2012). Avian species communities and predator–prey interactions may be modified based upon adjacent land cover type, which can influence predator density and type, and decrease the stability of population dynamics (Kareiva, 1987; Rosenzweig & MacArthur, 1963). The synergistic effects of changes in land cover and flooding regimes on tidal marsh community interactions require further study to improve vulnerability estimates for species of concern. Our aim was to assess how inundation regime influences avian predator (raptors, ardeids, and scavengers) behavior.

**Figure 1:**
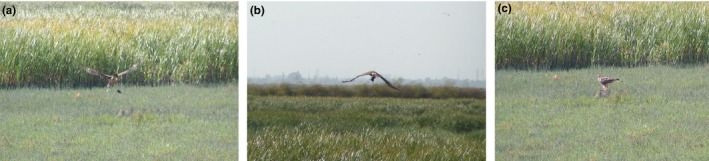
Northern Harriers (*Circus hudsonius*) prefer wide‐open habitats with low vegetation to course low over the ground to locate and capture prey. (a) A female Northern Harrier flies low over salt marsh while hunting and aerial dives to capture small prey, (b) A small mammal is carried to a feeding spot, and (c) A feeding perch on a low post in salt marshes. Photo credit: Brooke Hill, USGS

The premise of our study was to use the natural seasonal variation in lunar tidal cycles to measure whether predator foraging behavior changed with water levels in tidal marshes. We assumed that elevated water levels represent an analog for future high water conditions with climate change. Normally, high water levels are often associated with low pressure storms and are difficult to predict, necessitating our use of the natural tidal cycle for this study. We hypothesized that avian predators would increase their presence and activity during high tides, when increased water levels across the tidal habitats increase vulnerability and availability of prey (Figure 1, e.g., mice, voles, rails, aquatic species). In this paper, we present evidence that tidal inundation patterns and time of day or year affect the presence, abundance, and behavior of the avian predators within tidal marshes. We enumerate “predation pressure” defined as the combined probability of predator presence related to water level.

## 2 MATERIALS AND METHODS

### 2.1 Study areas

San Francisco Bay estuary supports one of the largest expanses of tidal marsh on the Pacific coast of North America, USA, but has been heavily fragmented due to human use and land conversion resulting in many wildlife species of conservation concern. The pickleweed‐dominated (*Salicornia pacifica*) tidal marsh is important habitat for sensitive and secretive salt marsh‐dependent wildlife species, including the federal and state endangered California Ridgway's rail (*Rallus obsoletus obsoletus*; hereafter, Ridgway's rails), federal and state endangered salt marsh harvest mouse (*Reithrodontymys raviventris*), and state‐threatened California black rail (*Laterallus jamaicensus coturniculus*). Compared to tidal wetlands of the Atlantic or Gulf coasts of North America, the Pacific coast salt marshes are topographically isolated by rugged coastlines or fragmented by human development (Josselyn, 1983; Zedler, 1982). Tidal marshes experience daily and seasonal semidiurnal tidal regimes and are subject to storm surges in the winter, particularly during El Niño ocean conditions and atmospheric river storms (Barnard et al., 2017; Thorne et al., 2012).

**Figure 2:**
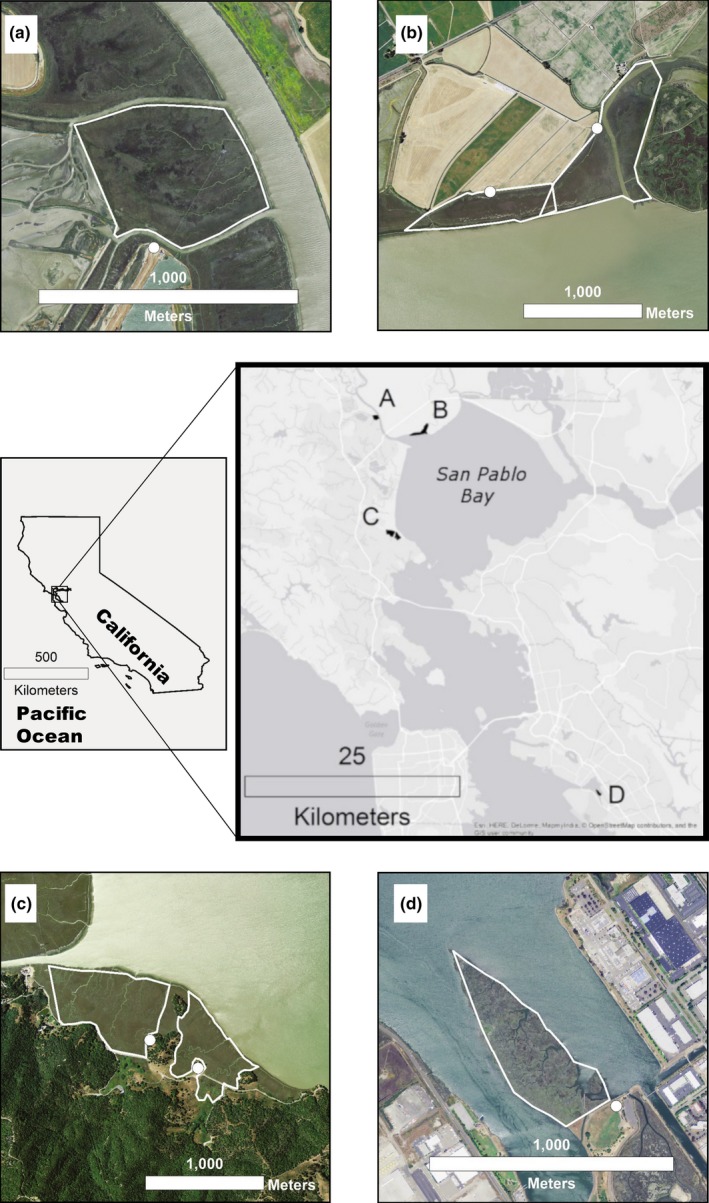
Tidal marsh study sites—(a) Black John marsh, (b) Tolay Slough, (c) China Camp State Park, and (d) Arrowhead marsh. Sites were surveyed from September 2010 to February 2011 in the San Francisco Bay estuary, California, USA. Dots represent the observation points for the predator surveys. All study sites are fully tidal and have different adjacent land cover types

This study was conducted at four tidal marshes located adjacent to different land cover types common to many estuaries (Figure 2, Table 1; Arrowhead, Black John, China Camp, and Tolay Slough). Arrowhead, the smallest study site, occurs within the dense urbanized area of Oakland on San Francisco Bay. With the exception of a small portion of uplands to the southeast, it is surrounded by open water. Cordgrass species (*Spartina* sp.) dominate the low, planar elevations that are more frequently inundated—ideal habitat for the Ridgway's rail. In contrast, steep slopes of undeveloped bay‐oak woodland surround China Camp State Park (hereafter China Camp) which is located on the western shore of San Pablo Bay. Tolay Slough is within the western arm of San Pablo Bay National Wildlife Refuge along the northern shore of San Pablo Bay and adjacent to agricultural fields that have subsided since being cut off from natural tidal fluctuation more than a century ago. Black John borders the Petaluma River, a tributary to northern San Pablo Bay, and is adjacent to a large tidal restoration. China Camp, Tolay Slough, and Black John provide habitat for both the salt marsh harvest mouse and California black rail with lower incidences of Ridgway's rail, indicating that these sites support a larger habitat gradient within the tidal frame.

**Table 1:** Descriptive information for the tidal marsh study sites in San Francisco Bay, California

Marsh	Area (ha)	Mean Elevation (m)*	Elevation Range (m)*	MHHW (m)*	MSL (m)*
Arrowhead	17	1.6	0.37	1.91	1.03
Black John	31	1.8	0.45	1.91	1.06
China Camp	97	1.8	0.55	1.95	1.02
Tolay Slough	90	2.0	1.49	1.85	1.04

* Indicates that elevations, tide datums, and elevation values are found in Takekawa et al. (2013). Meters (m) are relative to NAVD88.

### 2.2 Marsh flooding characterization

Water level loggers (WLL; Solinst, Georgetown, ON, Canada) were deployed continuously at the mouth of a main channel at each study site to capture local hydrograph information to quantify high and low water levels. WLL units were surveyed with a Leica RX1200 Real‐Time Kinematic (RTK) Global Positioning System (GPS) rover (±2 cm *x*, y, *z* accuracy; Leica Geosystems, Inc., Norcross, GA) at the time of deployment and during every data download to correct for movement of the WLLs. The rover positions were referenced to the Leica Smartnet system (http://smartnet.leica-geosystems.us) and to a benchmark (X 552 1956 Mare Island) to ensure the vertical accuracy of the survey. Water levels were measured at 6‐min intervals and were corrected for local barometric pressure.

To obtain elevations of the marsh platform for each site to calculate flooding levels, we used previously collected ground elevation surveys from Takekawa et al. (2013). The surveys were conducted between 2008 and 2010 with a Real‐Time Kinematic (RTK) GPS rover. We presented the inundation state of the tidal marshes as water levels relative to mean tidal marsh elevation.

### 2.3 Avian predator surveys

Paired surveys were conducted semimonthly from September 2010 to January 2011, once at the predicted monthly high tide (“high‐tide surveys”; National Oceanic and Atmospheric Administration http://tidesandcurrents.noaa.gov) and one week later during a diurnal low tide (“low‐tide surveys”); temporal separation of the high‐ and low‐tide surveys was necessary due to daylight and surveyor time constraints. In the San Francisco Bay region, diurnal monthly high tides from September to January are high enough to flood the tidal marsh platform and produce the highest daytime tides of the year, and therefore, these months were selected for the surveys. High‐tide surveys started at the first diurnal low tide and continued through the high tide within a single day (8–10 hr). High‐tide and low‐tide surveys began at sunrise if the first low or high tide of the day occurred pre‐dawn and continued to the next low or high tide. Surveys lasted the entire tidal cycle (>8/hr) for that sample day to control for other factors that may influence predator activity, such as time of day.

We focused our surveys on any avian predators observed during the survey period, and we did not differentiate if predators were targeting aquatic or terrestrial prey. We grouped red‐tailed hawks, white‐tailed kites, northern harriers, peregrine falcons, kestrels, and ospreys as raptors. We grouped red‐tailed hawks (*Buteo jamaicensis*), white‐tailed kites (*Elanus leucurus*), northern harriers (*Circus hudsonius*), peregrine falcons (*Falco peregrinus*), American kestrels (*Falco sparverius*), and ospreys (*Pandion haliaetus*) as raptors (Supplemental Table S1). Great blue heron (*Ardea Herodias*), great egret (*Ardea alba*), and snowy egret (Egretta thula) were grouped as ardeids, while gulls (*family ‐ Laridae*) and turkey vultures (*Cathartes aura*) were grouped as scavengers. Gulls were the only birds not identified to species during observations (Table S1). All individuals recorded had to be observed within each marsh patch area during the observation period, individuals could have been roosting, walking, or flying within the study site to be counted.

For each high‐tide survey at Arrowhead and Black John, teams of two observers were stationed at a predetermined vantage point with binoculars and a spotting scope. Because China Camp and Tolay Slough had a site configuration that obscured complete visual coverage, two teams of observers counted birds with binoculars and spotting scopes at two distinct vantage points at each site. During the paired low‐tide surveys, we assumed only one observer was needed to cover each site due to the elevated vantage point, small number of avian predators observed, and low‐activity levels.

Each observer team conducted alternating instantaneous scan surveys and focal observation surveys every 12 min (e.g., 09:00 = scan, 09:12 = focal, 09:24 = scan, 09:36 = focal) throughout the survey period (Altmann, 1974). For the instantaneous scan surveys (*n* = 792, LT = 398, HT = 394), we recorded the beginning and end time, number of individuals of each avian predator species, and activity of each individual [e.g., roosting on a man‐made or natural structure, flying, feeding, walking, or perched on the ground (Hancock, Kushlan, Gillmore, & Hayman, 1984)]. For the focal observation surveys (*n* = 793, total time = 8,568 min; *n* = 511 and 5,399 min during HT, *n* = 307 and 3,169 min during LT), we recorded the beginning and end time, species, and individual number of foraging attempts or “strikes”. The number of successful strikes was defined by either the presence of a captured prey item or feeding behavior which also was recorded. If more than one predator was present at the time of a focal observation, we used randomly generated compass bearings to choose an individual. Captured prey items were identified when visible. Because foraging behavior differs among avian predators, we also quantified effort by the bird between strikes; for example, the number of steps taken by ardeids. No mammalian predators were observed during the study, and since surveys were conducted during daylight hours, no owls (*Strigidae*) or other nocturnal predators were observed.

### 2.4 Data analysis

We calculated the mean (±*SE*) water level for each survey period and site and analyzed differences in inundation across sites and survey periods with an analysis of variance and a Tukey honest significant difference test (TukeyHSD). We quantified the numerical response of avian predators to high and low tides, limiting analysis to a ±2‐hr window around the minimum or maximum water level to isolate the tidal effect, and calculated the mean number of predators per scan. We calculated the proportion of species and predator guilds at each site during high‐ and low‐tide surveys. Thus, we quantified the effect of water level on diversity and number of predator species and groups.

To assess the behavioral response to water level, we calculated the strike and success rate by predator guild by site and tide survey. A strike or success was defined as an observation of captured prey or feeding by predators. For this analysis, only white‐tailed kites and northern harriers were included as raptors, and only great egrets and great blue herons as ardeids. The number of successes (and strikes) was summed by guild and divided by the total number of minutes spent observing individuals from that guild, across all the surveys.

We then evaluated the numerical response of predators to water level on the marsh. We used a zero‐inflated Poisson model to predict the number of raptors and ardeids, with all available data from the scans. Zero‐inflated Poisson models use a logistic distribution to first predict presence or absence of a predator, then use a Poisson distribution to model the number of predators. We considered covariates for flooding stage (flood, ebb), tide (low, high), month (a surrogate for season), time of day (morning [<11:00 am], noon [11:00 a.m.–2:00 pm], afternoon [>2:00 pm]), and water level (relative to mean marsh elevation).

The best‐supported model for raptors and ardeids was selected based on Akaike's Information Criterion (AIC, Burnham & Anderson, 2002). We then calculated the numerical response of predators to water level using the model‐estimated coefficients for water level and a transformation to convert log‐odds from the logistic regression model to probability. We multiplied this probability by predicted counts from the Poisson regression models to calculate an estimate of raptor and ardeid responses to water level while controlling for important covariates.

## 3 RESULTS

### 3.1 Marsh tidal inundation

Mean elevation for tidal marsh study sites ranged from 1.6 to 2.0 m (NAVD88) and was used to determine level of water relative to the marsh surface for each survey. Measured water levels were higher during high‐tide surveys compared with low‐tide surveys (*F*_1,43_ = 108.27, *p* < 0.001). Water levels during the high‐tide surveys in December and January were significantly higher than those in September and October (TukeyHSD, *p* < 0.001), largely due to differences in the seasonal tidal cycle patterns (Figure 2).

### 3.2 Avian predator composition

**Figure 3:**
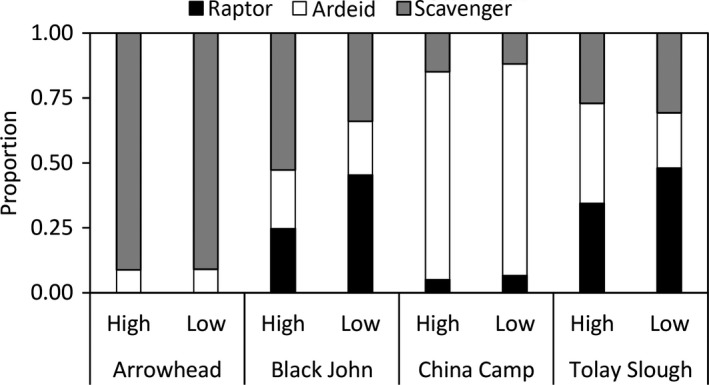
Relative proportion of each predator guild by site and tide survey across the entire study period. Data are from scans that were within 4‐hr window around high (low) tide

**Figure 4:**
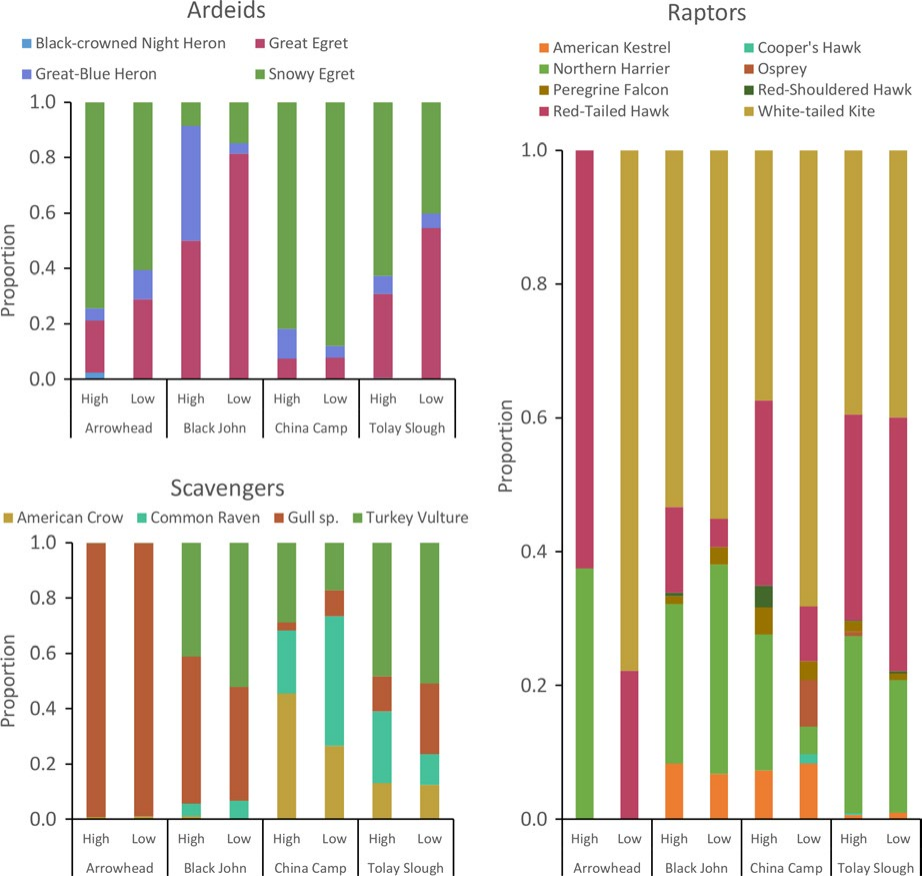
Predator species composition by tidal marsh study site and by tide survey across the study period

Sixteen avian predator species were observed from September 2010 to February 2011. However, we recorded only nine of the 16 (56%) species present across all four sites. Arrowhead had the lowest species richness and China Camp the highest, with 10 and 18 species observed (Figure 3 and 4). Although scavengers were counted most frequently (Table 2), they were never observed foraging in the marsh and therefore were excluded from water level response analyses.

**Table 2:** Mean counts of avian predators per scan within a ± 2‐hr window of high or low tide from September 2010 to February 2011 across all study sites (n is the total number of scan surveys)

Site	High‐tide surveys					Low‐tide surveys				Total
	*n*	Ardeids	Raptors	Scavengers	Total	*n*	Ardeids	Raptors	Scavengers	
Arrowhead	60	8.21	0.08	90.41	98.72	59	4.12	0.15	48.92	53.19
Black John	64	0.86	2.00	1.86	4.72	58	0.29	1.10	0.52	1.91
China Camp	64	9.97	1.33	1.95	13.25	61	6.33	0.62	0.64	7.59
Tolay Slough	64	4.75	8.95	3.80	17.5	66	1.03	4.89	1.51	7.44

We found that on average across all sites there were significantly more raptors observed during high‐tide scans than during low‐tide scans (*F*_3,1_ = 6.37, *p* = 0.015), while the number of ardeids per scan was marginally greater during high‐tide scans (*F*_3,1_ = 3.15, *p* = 0.083; Table 3). The number of scavengers per scan was not significantly different between tide surveys (*F*_3,1_ = 2.11, *p* = 0.15). China Camp had the highest relative densities of ardeids among sites, while Black John and Tolay Slough had highest relative densities of raptors (Figure 3), and most scavengers were observed at Arrowhead. Red‐tailed hawks, white‐tailed kites, and northern harriers were the most frequently observed raptor species during both high‐ and low‐tide surveys (Figure 4). During the 4‐hr window around low tide, no raptors were observed at Arrowhead and no raptors or ardeids were seen at Black John. However, white‐tailed kites, red‐tailed hawks, or northern harriers were seen during the 4‐hr window around high tide across all sites (Figure S1).

**Table 3:** Zero‐inflated Poisson model predictors for ardeids include study sites, water level, and time

	Estimate	*SE*	*Z* * value
Poisson predictors			
Intercept***	−0.25559	0.0729	−3.506
Black John***	−2.72295	0.15309	−17.787
China Camp***	−1.62325	0.05352	−30.331
Tolay Slough***	−2.24254	0.07098	−31.595
Low‐Tide Survey***	−0.56069	0.08097	−6.925
Water Level***	0.38806	0.07309	5.309
Water Level^2^ ***	−0.19127	0.06109	−3.131
September***	−0.94791	0.05826	−16.272
October***	−0.96521	0.06346	−15.21
November***	−0.60084	0.05571	−10.785
December***	−0.35279	0.05052	−6.983
Black John: low‐tide.survey#	0.67657	0.35017	1.932
China Camp: low‐tide.survey**	0.2414	0.09068	2.662
Tolay Slough: low‐tide.survey	−0.02368	0.13674	−0.173
Binomial predictors			
Intercept***	−5.7057	0.5797	−9.842
Black John	0.5443	0.6334	0.859
China Camp**	−1.3347	0.4814	−2.772
Tolay Slough**	−1.6542	0.5909	−2.799
Low‐tide survey	0.2485	0.6031	0.412
Water level**	−0.8314	0.2854	−2.913
Water level^2^ *	0.7457	0.3042	2.451
Morning*	0.8328	0.334	2.493
Afternoon	0.1973	0.3529	0.559
Black John: low‐tide survey*	1.7706	0.8232	2.151
China Camp: low‐tide survey#	1.1122	0.635	1.751
Tolay Slough: low‐tide survey*	1.6322	0.7299	2.236

Note

Significance codes: 0 ‐ ^***^ ‐ 0.001 ‐ ^**^ ‐ 0.01 ‐ ^*^ ‐ 0.05 ‐ ^#^ ‐ 0.1

### 3.3 Predator behavior

**Figure 5:**
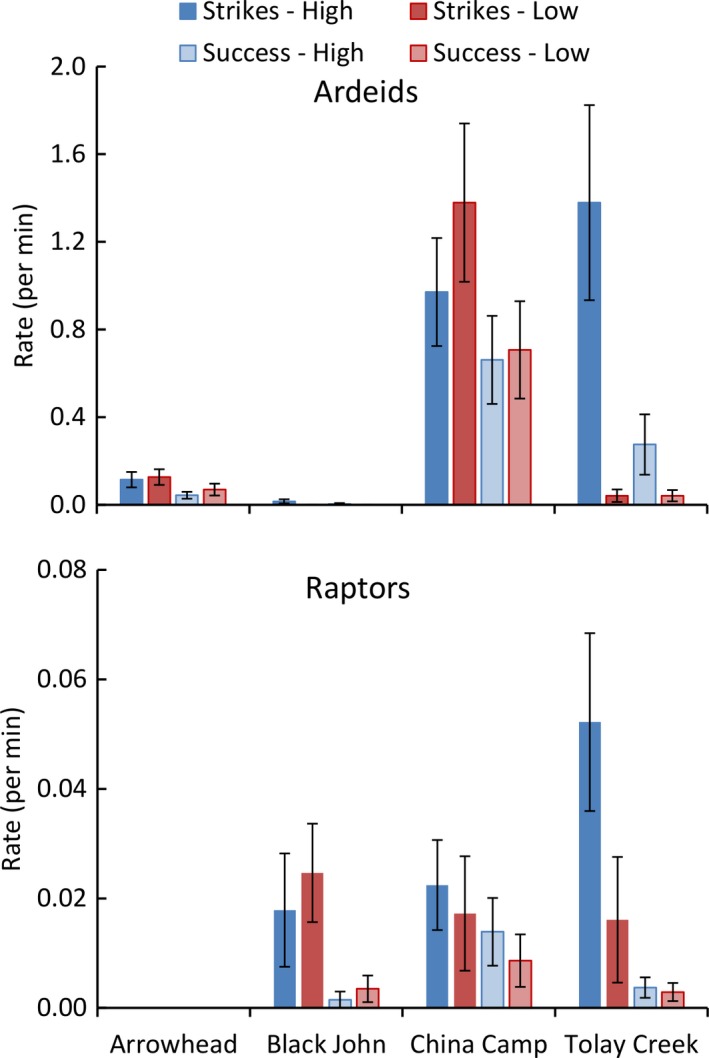
Strike and success rates (observations/total observation time) of ardeids and raptors across all sites and tide surveys during 793 focal surveys, lasting 8,568 min. Strikes were defined as a foraging attempt, and successful strikes were defined by either the presence of a captured prey item or feeding behavior. The rates of strikes and successes by raptors were typically an order of magnitude less than the rates by ardeids

**Figure 6:**
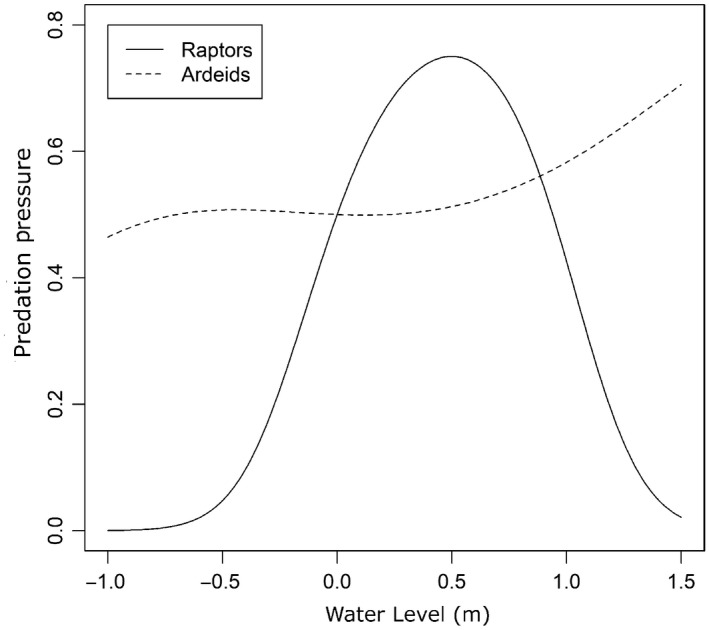
Numerical response of predators to water level (m), relative to mean marsh elevation. Zero‐inflated Poisson models were used to predict the response of raptor and ardeid density to changes in water level, while controlling for covariates such as season, time of day, and tide stage. The probability of a predator being present from the logistic regression model was multiplied by the predicted number of predators from the Poisson regression model

Study sites varied in the rate of strikes and successful strikes, with most effort occurring during high tides (Figure 5); however, the large intersite variation and low replication (*n* = 4) precluded significant results in analysis with one‐way ANOVA. For ardeids, the rate of strikes on high tides at Tolay Slough and Black John tended to be higher than strikes at low tides, while the rate of strikes on high tides tended to be lower than strikes at low tides at Arrowhead and China Camp (Figure 6). For raptors, more strikes were observed at high tide at Tolay Slough and China Camp, and high tide capture rates were highest among raptors at China Camp (Figure 6).

Raptors were seen at Arrowhead, but there was no hunting observed (successful strikes or not) during our surveys. Ardeids were numerous, reflecting Arrowhead's lower relative elevation in the tidal frame and higher frequency of flooding. Meanwhile, ardeids at Black John were never recorded to have attempted or successfully captured prey during low tides but were observed to have a single success during high tides. At China Camp, ardeids showed minimal difference in activity or success between tides; however, raptors demonstrated noticeable differences between high and low tides. Strike attempts were 2.4 times greater and successful captures 2.0 times greater during high‐tide events, and China Camp also had the highest success rate of the sites. Finally, both ardeids and raptors showed increased activity and efficiency at Tolay Slough during high‐tide events. Compared to low‐tide observations, ardeids at Tolay Slough demonstrated a 29‐fold increase in the number of capture attempts, and 4.4 times greater success rate when compared with other study sites. At Tolay Slough, raptors attempted strikes 6.2 times more during high‐tide events representing the highest activity rate recorded by raptors at any site, but there was no difference in their rate of strike success (Figure 6).

### 3.4 Water level response

The top zero‐inflated Poisson model for raptors included predictors for site, water level, survey tide, month, and tidal stage, while the top model for ardieds was similar except that time of day replaced tidal stage (Tables 3, 4, Tables S2, S3). Low‐tide surveys had significantly fewer raptors and ardeids than the high‐tide surveys. There were significantly more raptors in December and January than the other months and more ardeids in January. Ardieds were more prevalent in the morning than mid‐day or afternoon, while raptors did not exhibit a significant response to time of day. Model results from the 792 scans showed that the raptor response to water level was unimodal with a peak at a depth of 0.5 m over the marsh platform, while ardieds responded positively to increasing water level (Figure 5).

**Table 4:** Zero‐inflated model predictors for raptors

	Estimate	*SE*	*Z* * value
Poisson predictors			
Intercept***	−2.63148	0.09457	−27.826
China Camp***	−1.55942	0.095135	−16.392
Tolay Slough***	0.434076	0.067916	6.391
Low‐tide survey**	−0.2004	0.070222	−2.854
Water level**	0.192848	0.060024	3.213
September***	−1.43772	0.105765	−13.594
October***	−0.56732	0.074203	−7.645
November***	−0.28937	0.074129	−3.904
December	0.001196	0.061871	0.019
Stage ebb	0.046496	0.050437	0.922
Binomial predictors			
Intercept***	−8.392	1.0269	−8.172
Water level**	3.6528	1.1829	3.088
Water level2#	−4.266	2.5454	−1.676
Low‐tide survey**	2.5176	0.9476	2.657

Note

Significance codes: 0 ‐ ^***^ ‐ 0.001 ‐ ^**^ ‐ 0.01 ‐ ^*^ ‐ 0.05 ‐ ^#^ ‐ 0.1

## 4 DISCUSSION

### 4.1 Community ecology

Each marsh site hosted a unique community of native avian predators, likely influenced by the nuances of site characteristics and position within the surrounding landscape. Consequently, the observed predator–prey interactions differed between sites, illustrating the complexity of studying community composition and interactions. For example, at Tolay Slough, the re‐occurring presence of red‐tailed hawks, a nontraditional marsh predator (Johnston, 1956; Page & Whitacre, 1975), was likely a result of the adjacent agricultural fields and nearby power line poles that can serve as roosts (Knight & Kawashima, 1993). The large number of scavengers such as gulls observed at Arrowhead marsh are attributable to nearby urban development (e.g., parking lots, dumps, housing; Vermeer, Power, & Smith, 1988), scavengers have been shown to opportunistically forage on the eggs and nestlings of protected species (e.g., Ridgeways rail; USFWS, 2013); however, none were observed foraging in the marsh during our study. The overall high diversity of raptors observed at China Camp may be related to the adjacent oak woodland habitats (Takekawa et al., 2011).

Human development and restoration actions may enhance the habitat availability and foraging access of predator species. For example, features such as levees have led to colonization of plant species favorable for roosting (e.g., coyote brush, eucalyptus groves), and within close proximity to abundant prey resources in flooded marshes (Tsao, Takekawa, Woo, Yee, & Evens, 2009). Additionally, artificial structures (e.g., powerlines, old abandoned structures, fence lines) create roosting habitats for several of the species observed in this study. A study of white‐tailed kites showed that individuals achieved the highest foraging efficiency using the hover and strike method of hunting as compared to roosting; however, they were observed roosting on powerlines, polyvinyl chloride (PVC)‐markers, old wooden channel marker signs, and old fence posts, which were used as perches preceding a strike attempt within the marsh (Tarboton, 1978). Thus, adjacent land cover and human modifications influence the predator–prey response to increased flooding levels.

The fragmented nature of marsh habitats in estuaries has reduced the patch size for terrestrial wildlife (Fahrig, 2003). Our results confirmed that predation pressure for tidal marsh species within these small patches increased with increased flooding during high tides. Patchy tidal marsh habitats result in lower dispersal of resident terrestrial species between neighboring sites to escape flooding and predators, and this has been shown to amplify environmentally driven bottlenecks in three subspecies of song sparrows (*Melospiza melodia*) in the San Francisco estuary (Marshall, 1948). Raptors have been shown to be the primary predator of the endangered California Ridgeway's rails (Casazza et al., 2017). The extensive restoration efforts currently underway across this estuary provide an opportunity to reconnect fragmented habitats and boost prey populations, although there is a substantial time lag before restored areas can provide suitable habitats (Whittingham & Evans, 2004). Consideration of such landscape factors is important when evaluating marsh vulnerability to flooding and responses of avian predators when assessing restoration sites and conservation planning.

### 4.2 Tidal flooding

**Figure 7:**
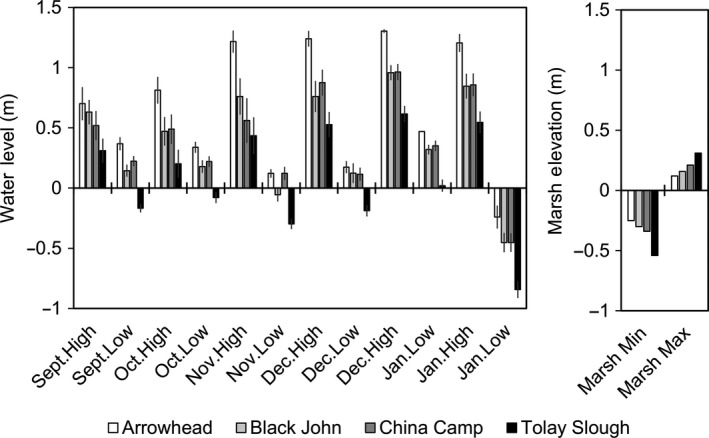
Observed mean (±*SE*) water level (m) by survey period and minimum and maximum marsh elevation (m) relative to average marsh elevation, across each site

When attempting to understand how ecosystems may change with climate change and sea‐level rise, it can be informative to evaluate analoges (Fernández, Hamilton, & Kueppers, 2015; Kellet, Hamilton, Ness, & Pullen, 2015). In estuaries, seasonal tidal flooding patterns can provide an analog of future flooding conditions and insight into predator behavioral changes with increased water levels. For example, in our study, we measured water levels 0.5–1.4 m above mean tidal marsh elevation (Figure 7), with the highest measured water levels between December and January. Those particular times of year with higher flooding levels can serve as the best analog of future sea levels, with current projections for this region 0.5–3.0 m above the 1991–2009 mean by 2100 (Griggs et al., 2017). Incorporating effects of tidal flooding on predator–prey relationships illustrates the importance of assessing multiple stressors to understand the vulnerability of wildlife communities of management concern. Increased predation on elevated high tides could create a conservation concern for protected species, but the loss of prey species may also have a cascading effects resulting in loss of native avian predators (Pichegru, Ryan, Crawford, Lingen, & Gremillet, 2010), or facilitating the introduction of invasive species (Needles, Gosnell, Waltz, Wendt, & Gaines, 2015). Here, we focused on avian predators during tidal cycles to document predation pressure on terrestrial tidal marsh species, and we confirmed that predator number and activity increased during high‐tide events and predation pressure was lower during low tides. We also found that there was higher predation pressure from ardeids than raptor species with the exception of Tolay Slough, which was dominated by raptors. Many of the raptors in this region are migratory with different peaks of occurrence during fall migration (mid‐August through mid‐December). From 1985 to 2009, peak sightings were an order of magnitude higher and occurred later in the year for northern harriers (12.2 sightings per day on 4 November) compared to white‐tailed kites (1.2 sightings per day on 21 October; http://www.parksconservancy.org/assets/conservation/plants-and-animals/pdfs/ggro-timing-graphs.pdf).

Our results suggested that increases in flooding with sea‐level rise may increase predation pressure on terrestrial tidal marsh species, with unknown consequences to aquatic prey species. Kent (1986) found that fish species were the largest number of prey consumed by snowy egrets and herons in a Florida estuary, illustrating the importance of this food source with increased flooding of the wetland ecosystems. Flooding surges from atmospheric storms, wind and wave run up, and precipitation could further increase flooding levels when paired with increasing sea levels (Griggs et al., 2017; Wahl et al., 2017). For example, the El Niño oceanographic forcing and storms of 2015–2016 increased coastal flooding in California beyond any historic records (Barnard et al., 2017). Future projections of storm frequency and magnitude for the 21st century are variable, but a recent study suggests a potential doubling of extreme El Niño events (Cai et al., 2014). Increased predation pressure from sea‐level rise and storms could be a selective force affecting the presence and viability of prey populations given a possible increased risk from predation (Chevin & Hoffmann, 2017; Scharnweber et al., 2013). Our observations may be used as an analog for future conditions and illustrate the importance that predator pressure may play with changing inundation.

Despite the ability of tidal marshes to build elevation relative to sea‐level rise (Kirwan et al., 2016; Morris et al., 2016; Morris, Sundareshwar, Nietch, Kjerfve, & Cahoon, 2002), the daily, monthly, and yearly inundation patterns from flooding could increase the vulnerability of obligate marsh species of management concern and increase foraging opportunities for predators. In addition, terrestrial species may be synergistically affected by other stressors from flooding such as drowning and nest loss (Field et al., 2017; Hunter, 2017), highlighting the complexity of understanding the full effect of climate stressors on wildlife. Our study highlights the importance of predator–prey interactions and the amplification of predation pressure under flooded conditions, which has implications for population persistence in small, fragmented habitats under sea‐level rise. We conclude that focusing solely on habitat gains and losses from sea‐level rise while ignoring species interactions that include predator–prey dynamics within the habitat, may underestimate impacts to tidal marsh wildlife persistence.

## CONFLICT OF INTEREST

None declared.

## AUTHORS’ CONTRIBUTIONS

KT, KS, and JT conceived the project hypothesis and designed the methodology; KS, KT, and KB collected the data; KT, KS, KB, and JR analyzed the data. KT, KB, and JR led the writing of the manuscript. All authors contributed critically to the drafts and gave final approval for publication.

## Data Availability

All data needed to evaluate the conclusions in the paper are present in the paper. All raw data are archived at the US Geological Survey Science Base Catalog (http://www.sciencebase.gov/catalog/).
