# Supplementary material for: Flooding regimes increase avian predation on wildlife prey in tidal marsh ecosystems
Source: Ecol Evol. 2019 Jan 13;9(3):1083–94. doi: 10.1002/ece3.4792 (PMC6374721; doi:10.1002/ece3.4792)
Supplement: Supplementary file 1 [file ECE3-9-1083-s001.docx]

**Supplemental Information**

**
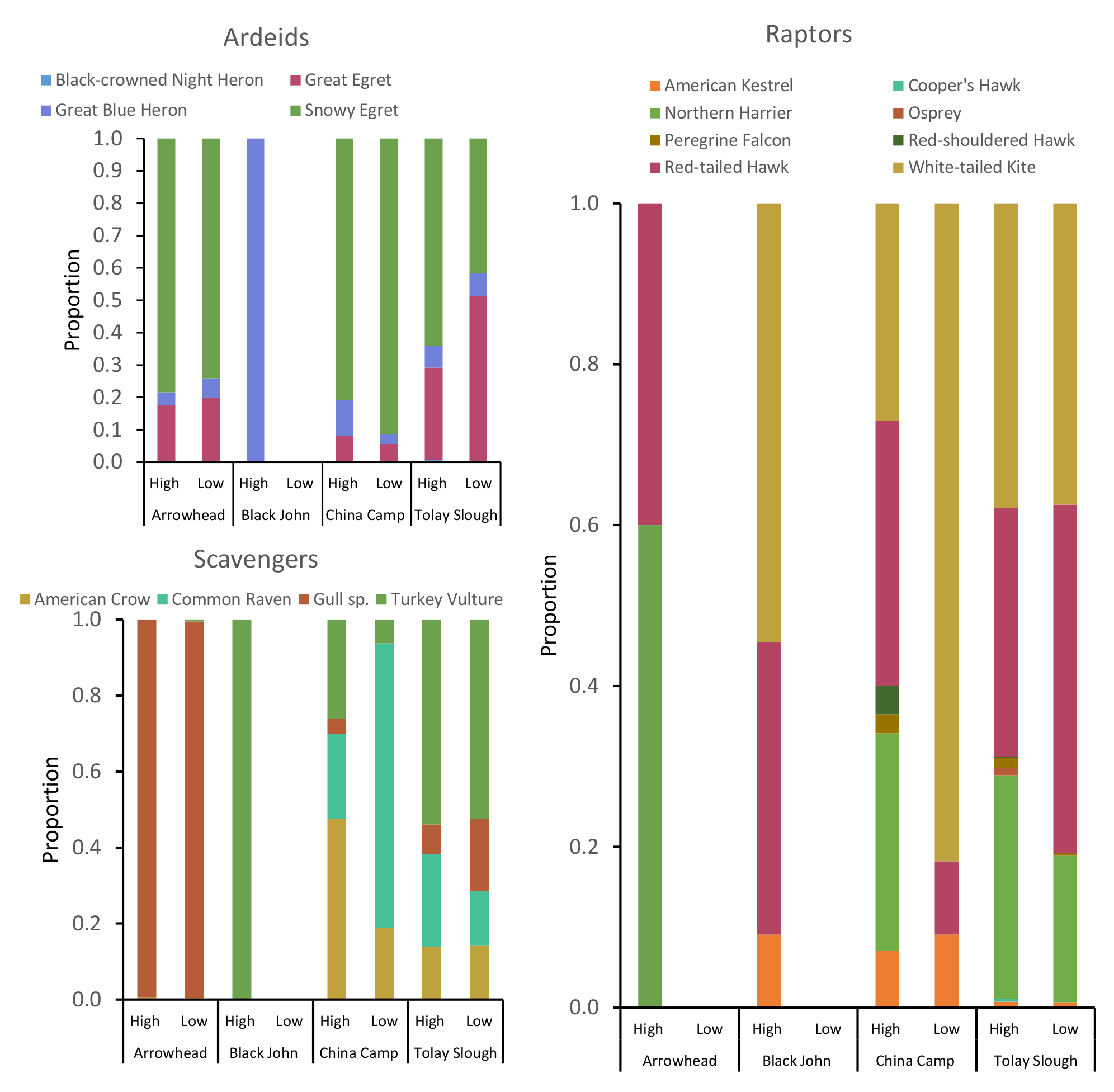
**

**Supplemental Figure 1.** Avian predator species observed during a 4-hour peak high tide window for the entire study period.

**Supplemental Table 1.** Marsh avian predators can prey upon terrestrial and aquatic species.

| Predator Species | Dominant Prey | References |
| --- | --- | --- |
| Black-crowned Night -Heron | Fish, insects, mammals | Henny et al. 2002 |
| Great Egret | Fish | Schlorff 1978 |
| Great-blue Heron | Fish | Hom 1983 |
| Snowy Egret | Small annelids, crustaceans, insects, and fish | Hom 1983 |
| American Crow | Insects, plant matter, birds, eggs, mammals, reptiles | Kalmbach 1939b |
| Common Raven | Arthropods, amphibians, reptiles, birds (adults, chicks, and eggs), small mammals, carrion, grains, buds, and berries. | Boarman and Heinrich 1999 |
| Gull Sp. (e.g., Western gull) | Fish, marine invertebrates, garbage | Pierotti and Annett 1995 |
| Turkey Vulture | Carrion | Kirk and Mossman 1998 |
| American Kestrel | Insects and rodents | Smallwood and Bird 2002 |
| Osprey | Fish | Henny et al. 2003 |
| Red-tailed Hawk | Mammals | Fitch et al. 1946a |
| Cooper’s Hawk | Birds | Fitch et a. 1946b |
| Peregrine Falcon | Birds | White et al. 2002 |
| White-tailed Kite | Mammals | Dunk 1995 |
| Northern Harrier | Mammals | Bildstein 1987 |
| Red-Shouldered Hawk | Mammals | Dykstra et al. 2008 |

**Supplemental Table 2.** AIC table for zero-inflated models of raptors based on the scan data. Models on the left of the | were regressed with a Poisson distribution, while models on the right were fit using logistic regression.

| Models | k | Resid. Df | Log-likelihood | dAIC | weight |
| --- | --- | --- | --- | --- | --- |
| offset(log(Area)) + Site + Tide.Survey + WaterDepth + Month + Stage \| offset(log(Area)) + WaterDepth + WaterDepth^2^ + Tide.Survey | 13 | 606 | -1110.83 | 0 | 0.45 |
| offset(log(Area)) + Site + Tide.Survey + WaterDepth + WaterDepth^2^ + Month \| offset(log(Area)) + Tide.Survey + WaterDepth + WaterDepth^2^ | 13 | 606 | -1111.05 | 0.44 | 0.36 |
| offset(log(Area)) + Site + Tide.Survey + WaterDepth + Month \| offset(log(Area)) + Tide.Survey * Site + WaterDepth + WaterDepth^2^ | 16 | 603 | -1109.44 | 3.21 | 0.09 |
| offset(log(Area)) + ToD + Site + Tide.Survey + WaterDepth + WaterDepth^2^ + Month \| offset(log(Area)) + Tide.Survey + WaterDepth + WaterDepth^2^ | 15 | 604 | -1110.8 | 3.93 | 0.06 |
| offset(log(Area)) + Site + Tide.Survey + WaterDepth + WaterDepth2 + Month \| offset(log(Area)) + Tide.Survey * Site + WaterDepth + WaterDepth^2^ | 17 | 602 | -1109.13 | 4.6 | 0.04 |
| offset(log(Area)) + Site + WaterDepth + Month \| offset(log(Area)) + WaterDepth + WaterDepth^2^ | 10 | 609 | -1125.55 | 23.43 | 0 |
| offset(log(Area)) + Tide.Survey + WaterDepth + Month \| offset(log(Area)) + Tide.Survey + WaterDepth | 9 | 610 | -1463.72 | 697.78 | 0 |
| offset(log(Area)) + WaterDepth * ToD + Month \| offset(log(Area)) + WaterDepth + WaterDepth^2^ + ToD | 14 | 605 | -1482.47 | 745.27 | 0 |
| offset(log(Area)) + WaterDepth * ToD + WaterDepth^2^ \| offset(log(Area)) + WaterDepth + WaterDepth^2^ + WaterDepth^3^ | 10 | 609 | -1570.39 | 913.12 | 0 |
| offset(log(Area)) + WaterDepth * ToD \| offset(log(Area)) + WaterDepth + WaterDepth^2^ + ToD | 10 | 609 | -1577.94 | 928.21 | 0 |
| NULL | 1 | 618 | -1607.88 | 970.1 | 0 |

Top AIC=2249.66, df.null=618. ToD: Time of Day

**Supplemental Table 3.** AIC table for zero-inflated models of ardieds based on the scan data. Models on the left of the | were regressed with a Poisson distribution, while models on the right were fit using logistic regression.

| Models | k | Resid. Df | Log-likelihood | dAIC | weight |
| --- | --- | --- | --- | --- | --- |
| offset(log(Area)) + Site * Tide.Survey + WaterDepth + WaterDepth^2^ + Month \| offset(log(Area)) + Site * Tide.Survey + WaterDepth + WaterDepth^2^+ ToD | 25 | 766 | -2435.85 | 0 | 0.94 |
| offset(log(Area)) + Site * Tide.Survey + WaterDepth + WaterDepth^2^ + Month \| offset(log(Area)) + Site * Tide.Survey + WaterDepth + WaterDepth^2^ | 23 | 768 | -2441.35 | 7 | 0.06 |
| offset(log(Area)) + Site + Tide.Survey + WaterDepth + WaterDepth^2^+ Month \| offset(log(Area)) + Site + WaterDepth + WaterDepth^2^ + Tide.Survey | 17 | 774 | -2449.93 | 12.17 | 0 |
| offset(log(Area)) + Site + Tide.Survey + WaterDepth + WaterDepth^2^ + Month \| offset(log(Area)) + Site * Tide.Survey + WaterDepth + WaterDepth^2^ | 20 | 771 | -2447.04 | 12.37 | 0 |
| offset(log(Area)) + Site + Tide.Survey + WaterDepth + WaterDepth^2^ + Month \| offset(log(Area)) + Site + WaterDepth + Tide.Survey | 16 | 775 | -2456.14 | 22.58 | 0 |
| offset(log(Area)) + ToD + Site + Tide.Survey + WaterDepth + WaterDepth^2^ \| offset(log(Area)) + ToD + Site + Tide.Survey + WaterDepth + WaterDepth^2^ | 17 | 774 | -2624.39 | 361.09 | 0 |
| offset(log(Area)) + ToD + Site + Tide.Survey + WaterDepth \| offset(log(Area)) + ToD + Site + Tide.Survey + WaterDepth | 15 | 776 | -2631.09 | 370.47 | 0 |
| offset(log(Area)) + ToD + Site + WaterDepth \| offset(log(Area)) + ToD + Site + WaterDepth | 13 | 778 | -2672.97 | 450.25 | 0 |
| offset(log(Area)) + zstar + Site \| offset(log(Area)) + WaterDepth + Site | 9 | 782 | -2699.15 | 494.6 | 0 |
| offset(log(Area)) + Site \| offset(log(Area)) + Site | 7 | 784 | -2815.03 | 722.36 | 0 |
| offset(log(Area)) + Tide.Survey + WaterDepth + WaterDepth^2^ + Month + Stage \| offset(log(Area)) + Tide.Survey + WaterDepth + WaterDepth^2^ + Stage | 13 | 778 | -3290.92 | 1686.13 | 0 |
| offset(log(Area)) + Tide.Survey + WaterDepth + WaterDepth2 + Month \| offset(log(Area)) + Tide.Survey + WaterDepth + WaterDepth2 + ToD | 13 | 778 | -3338.51 | 1781.32 | 0 |
| offset(log(Area)) + WaterDepth * Stage + WaterDepth2 + ToD \| offset(log(Area)) + WaterDepth + WaterDepth2 + Stage | 10 | 781 | -3415.8 | 1929.89 | 0 |
| offset(log(Area)) + ToD \| offset(log(Area)) + ToD | 5 | 786 | -3716.44 | 2521.19 | 0 |
| offset(log(Area)) + Tide.Survey \| offset(log(Area)) + Tide.Survey | 3 | 788 | -3723.64 | 2531.58 | 0 |
| NULL | 1 | 790 | -3816.74 | 2713.77 | 0 |

Top AIC=4923.7, null df = 790. ToD = Time of day

**Supplemental References**

Bildstein KL. 1987. Behavioral ecology of Red-tailed Hawks (Buteo jamaicensis), Rough-legged Hawks (Buteo lagopus), Northern Harriers (Circus cyaneus), and American Kestrels (Falco sparverius) in south central Ohio. Ohio Biol. Surv. Biol. Notes no. 18.

Boarman WI. and Heinrich, B. 1999. Common Raven (Corvus corax), version 2.0. In The Birds of North America (A. F. Poole and F. B. Gill, Editors). Cornell Lab of Ornithology, Ithaca, NY, USA. <https://doi.org/10.2173/bna.476>

Dykstra CR., Hays JL., and Crocoll ST. 2008. Red-shouldered Hawk (Buteo lineatus), version 2.0. In The Birds of North America (A. F. Poole, Editor). Cornell Lab of Ornithology, Ithaca, NY, USA. https://doi.org/10.2173/bna.107

Dunk JR. 1995. White-tailed Kite (Elanus leucurus), version 2.0. In The Birds of North America (A. F. Poole and F. B. Gill, Editors). Cornell Lab of Ornithology, Ithaca, NY, USA. https://doi.org/10.2173/bna.178

Fitch HS., Swensen F, and Tillotson, DF. 1946a. Behavior and food habits of the Red-tailed Hawk. Condor no. 48:205-217.

Fitch HS., Glading, B., and House V. 1946b. Observations on Cooper's Hawk nesting and predation. Calif. Dep. Fish Game no. 32:144-154.

Henny CJ, Hill EF, Hoffman DJ, Spalding MG, Grove RA. 2002. Nineteenth century mercury: hazard to wading birds and cormorants of the Carson River, Nevada. Ecotoxicology 11:213–231

Henny CJ, Kaiser JL, Grove RA, Bentley VR, and Elliott JE. 2003. Biomagnification factors (fish to Osprey eggs from the Willamette River, Oregon, USA) for PCDDs, PCDFs, PCBs, and OC pesticides. Environ Monit Assess. Jun; no. 84 (3).

Hom CW. 1983. Foraging ecology of herons in a southern San Francisco Bay salt marsh. Colonial Waterbirds 6:37–44.

Kalmbach ER. 1939. The crow in its relation to agriculture. U.S. Dep. Agric. Farmers Bull. no. 1102.

Kirk DA and Mossman MJ. 1998. Turkey Vulture (Cathartes aura), version 2.0. In The Birds of North America (A. F. Poole and F. B. Gill, Editors). Cornell Lab of Ornithology, Ithaca, NY, USA. https://doi.org/10.2173/bna.339

Liebezeit JR. and George TL. 2002. Nest predators, nest-site selection, and nesting success of the Dusky Flycatcher in a managed ponderosa pine forest. Condor no. 104 (3):507-517.

Pierotti RJ and Annett CA. 1995. Western Gull (Larus occidentalis), version 2.0. In The Birds of North America (A. F. Poole and F. B. Gill, Editors). Cornell Lab of Ornithology, Ithaca, NY, USA. https://doi.org/10.2173/bna.174

Schlorff RW. 1978. Predatory ecology of the Great Egret at Humboldt Bay, California. In Wading Birds, edited by A. Sprunt Iv, J. C. Ogden and S. Winckler, 347-353. New York: Res. rep. no. 7. National Audubon Soc.

Smallwood JA and Bird DM. 2002. American Kestrel (Falco sparverius), version 2.0. In The Birds of North America (A. F. Poole and F. B. Gill, Editors). Cornell Lab of Ornithology, Ithaca, NY, USA. https://doi.org/10.2173/bna.602

White CM, Clum NJ, Cade, and Hunt WG. 2002. Peregrine Falcon (Falco peregrinus), version 2.0. In The Birds of North America (A. F. Poole and F. B. Gill, Editors). Cornell Lab of Ornithology, Ithaca, NY, USA. https://doi.org/10.2173/bna.660
